# Lysosomal Function and Axon Guidance: Is There a Meaningful Liaison?

**DOI:** 10.3390/biom11020191

**Published:** 2021-01-29

**Authors:** Rosa Manzoli, Lorenzo Badenetti, Michela Rubin, Enrico Moro

**Affiliations:** 1 Department of Molecular Medicine, University of Padova, 35121 Padova, Italy; rosa.manzoli@phd.unipd.it (R.M.); lorenzo.badenetti@phd.unipd.it (L.B.); michela.rubin@studenti.unipd.it (M.R.); 2 Department of Biology, University of Padova, 35121 Padova, Italy; 3 Department of Women’s and Children’s Health, University of Padova, 35121 Padova, Italy; 4 Pediatric Research Institute “Città della Speranza”, 35127 Padova, Italy

**Keywords:** axon guidance, lysosomal storage disorders, neuronal circuit

## Abstract

Axonal trajectories and neural circuit activities strongly rely on a complex system of molecular cues that finely orchestrate the patterning of neural commissures. Several of these axon guidance molecules undergo continuous recycling during brain development, according to incompletely understood intracellular mechanisms, that in part rely on endocytic and autophagic cascades. Based on their pivotal role in both pathways, lysosomes are emerging as a key hub in the sophisticated regulation of axonal guidance cue delivery, localization, and function. In this review, we will attempt to collect some of the most relevant research on the tight connection between lysosomal function and axon guidance regulation, providing some proof of concepts that may be helpful to understanding the relation between lysosomal storage disorders and neurodegenerative diseases.

## 1. Introduction

The development of the central nervous system (CNS) occurs during embryonic stages in a strictly temporally and spatially regulated manner, to allow for the organization of a network of nervous fibers that progressively increase the range of functional neuronal interactions. The high degree of complexity is achieved through a balanced and controlled process of axonal remodeling, followed by the formation of specific synapses that cross-connect target neuronal populations in order to establish a dynamic system of integrated communication [1,2]. Axonal remodeling involves the elimination of “useless” connections and the formation and growth of new dendritic spines and axonal trajectories that enable brain plasticity and correct sensory responses to external stimuli. Impaired axonal remodeling and pathfinding lead to defective synaptic connectivity and aberrant neuronal circuit function, which characterize both congenital disorders and neurodegenerative conditions. While we know which extrinsic factors (that is, environmental stimuli, injury, and neuronal activity) may govern the ability to increase the axonal branching and pruning [3], we do not have a clear picture of which intrinsic factors (genetically encoded proteins, type of cell population) finely modulate the overall setting of the neuronal network during early embryogenesis. In addition, we still lack extensive knowledge of whether and how in certain cases (for instance, brain injuries and traumatic insults) axonal regeneration takes place and which molecules control this process. Understanding the cascade of molecular events occurring during both embryonic brain development and after brain injury could allow for the identification of druggable targets that may hamper neurodegenerative conditions and prevent the onset of irreversible cognitive decline in certain inherited disorders. In the past few years, lysosomes have attracted a remarkable interest for their key role in the autophagic process during axonal remodeling [4]. Besides, many lysosomal enzyme defects have been detected in neurodegenerative conditions [5]. A few years ago, a pioneering study revealed a tight association between lysosomal activity and axonal pruning [6]. More recently, Farfel-Becker and colleagues demonstrated that lysosomes are actively delivered to the distal termini, suggesting their pivotal function in axonal dynamics [7]. Therefore, an emerging role of lysosomal activity in axon growth and guidance is gaining attention, positing lysosomes as one of the top interests of neurobiologists. In this review, we will try to briefly summarize the current knowledge of up-to-date discovered axonal guidance cues, providing an inferential nexus between their impaired activity and the brain pathogenesis of lysosomal storage disorders (LSDs).

## 2. Axonal Guidance Cues

**Figure 1:**
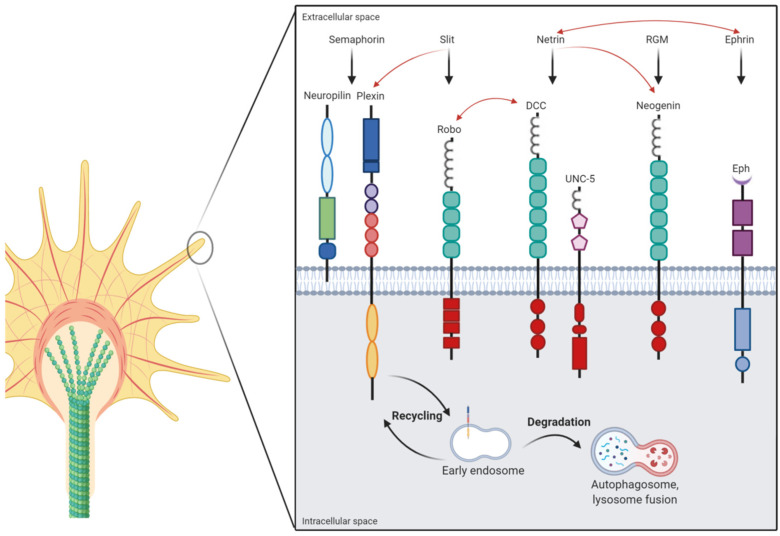
Axon guidance cues and endo-lysosomal pathway in the axonal growth cone. A schematic picture depicts the modular structure of major axonal guidance cue-related receptors and their respective ligands. Classical interactions are represented by black arrows, while red arrows indicate crosstalk between different axon guidance families. In the bottom part of the figure, the endosomal–lysosomal compartment is shown to mediate both receptor recycling and degradation. (created with BioRender.com).

The term “axon guidance” refers to all mechanisms that allow a developing axon to elongate from the neuronal soma and reach its target tissues. A nascent axonal growth cone can indeed integrate and transduce a multitude of different stimuli it receives from the surrounding extracellular environment. This results in precise and predictable shaping of axonal routes in the developing nervous system. The striking concept of axonal pathfinding has traveled along centuries, from Ramón y Cajal and his studies of the embryonic chick spinal cord (1890), to the identification and characterization of axon guidance cues’ major families (i.e., netrins, slits, semaphorins, and ephrins), together with morphogens, growth factors, glycoproteins, and cell adhesion molecules (CAMs) [8]. Axon guidance molecules can be divided into attractive and repulsive cues that act either diffusively over long distances or locally, in a contact-dependent manner. Cooperation between long-range and short-range guidance cues is required for the navigation of growing axons to their target cells (Figure 1).

### 2.1. Semaphorins

Semaphorins (SEMAs), first described in 1992 as “Fasciclin IV” by Kolodkin and colleagues, are a large family of proteins that can be either secreted, cell surface-attached, or membrane-bound [9]. Initially classified as repellents during axonal wiring, now it is known that some of them can also behave as attractants [10]. The growth-cone receptor PLEXIN is the most important protein involved in semaphorin signaling [11]. The interaction between semaphorin and PLEXIN can be direct or mediated by other membrane-associated proteins; for example, the SEMA3 class of semaphorin (except SEMA3E) interaction with PLEXIN is facilitated by neuropilins, type I transmembrane proteins located on the growth cone [12]. Cell adhesion molecules, such as Neuronal Cell Adhesion Molecule (Nr-CAM) and L1 cell adhesion molecule (L1-CAM) that associate with neuropilin receptors can be also required to mediate semaphorins’ effects and transduce SEMA3-dependent signaling [13]. Additional receptors that directly bind semaphorins include, for example, integrins [14] and proteoglycans [15]. Besides their role in axon guidance modulation, the SEMA3 family of proteins has been demonstrated to play an important function in vascular homeostasis also. In particular, SEMA3F seems to be involved in endothelial barrier homeostasis and monocyte migration [16]. Finally, semaphorins are known modulators of cancer cell behavior, such as glioblastoma cell growth, survival, invasiveness, and angiogenesis [17].

### 2.2. Ephrins

Ephrins were first discovered and described in the context of retinotopic mapping [18]. They are membrane-related guidance cues categorized into two classes: Ephrin-As (Ephrin-A1–Ephrin-A5), which are glycophosphatidylinositol (GPI)-anchored to the membrane, and Ephrin-Bs (Ephrin-B1–Ephrin-B3), which have a transmembrane domain followed by a short cytoplasmic domain [19]. Ephrin ligands bind to erythropoietin-producing hepatoma (Eph) receptors that represent the largest subfamily among receptor tyrosine kinases (RTK). Although ephrin-dependent signaling was initially thought to mediate chemorepulsive interactions, later evidence showed that both attractive and repulsive responses can occur [20]. Since ephrins are anchored ligands, their interaction with Eph receptors is allowed only at sites of cell–cell contact, so that ephrin signaling becomes fundamental in axon choice points, where axons select between two alternative routes [21]. Here, the recognition between ligand and receptor triggers a peculiar cascade known as “bidirectional signaling”; unlike the classical unidirectional model characterized by ligand-mediated receptor activation and a downstream signaling cascade inside the receptor-expressing cell, the Eph–ephrin interaction induces a response both in ligand and receptor-harboring cells. Thus, traditionally what happens inside the Eph-expressing cell is called the “forward signal” and depends on Eph kinase activity, while the term “reverse signal” refers to the events inside the ligand-bearing cell mediated by Src family kinases [22]. Due to the fact that ephrins are extracellular GPI-anchored proteins, they require a transmembrane protein to mediate reverse signaling. Indeed, it has been shown that Ephrin-A interacts with different co-receptors, such as the p75 neurotrophin receptor (NTR) [23], TrkB [24], and Ret [25].

### 2.3. Repulsive Guidance Molecule (RGM)

The Repulsive Guidance Molecule (RGM) was identified by Monnier and colleagues in 2002 while studying chick growth cones of retinal axons [26]. RGM is a GPI-anchored glycoprotein that has been proven to interact with neogenin (NEO) and acts as a repulsive guidance molecule [27]. Indeed, the NEO–RGM interaction is also pivotal for embryonic neurodevelopment, as it is required for neural tube closure and neuroepithelial polarization [28,29]. Interestingly, RGM has been shown to be involved in the invasion by inflammatory cells of the CNS during autoimmune encephalomyelitis, thus establishing a link between axonal guidance and neuroinflammation [30]. Another key aspect is that RGMs have been shown to inhibit Bone Morphogenetic Protein (BMP) signaling through the interaction with Growth Differentiation Factor 5 (GDF5), providing direct proof of the close connection between axonal pathfinding and morphogens activity [31].

### 2.4. Netrins

Netrins are a family of laminin-related proteins that act in the extracellular compartment as chemotropic guidance cues during neuronal development. In mammals, both secreted Netrin-1, 3, and 4) and membrane-tethered GPI-linked Netrins (Netrin-G1 and G2) have been discovered [32]. Unlike classical netrins, Netrin-G1 does not bind to known netrin receptors, but instead interacts specifically with the Netrin-G ligand (NGL1, also known as LRRC4c) to modulate neurite elongation and the laminar organization of dendrites and induce the accumulation of microglial cells around axons [33,34,35]. The history of netrins began in the early 1990s, starting with the description of *Caenorhabditis elegans* (*C. elegans*) genes unc-5 (UNC5 in mammals), unc-6 (NTN1 in mammals), and unc-40 (DCC and NEO in mammals, frazzled in *Drosophila melanogaster*) [36]. The UNC5 protein is implicated in the repulsive netrin-mediated axon guidance through heterodimerization with Deleted in Colorectal Cancer (DCC) for long-range repulsion, and with Down syndrome cell adhesion molecule (DSCAM) for short-range repulsion [37]. Given the homology with the UNC family, mammalian DCC, originally identified as a tumor suppressor, was first proposed as a mediator of netrin pathways in 1996 [38]. DCC is a transmembrane receptor of the immunoglobulin superfamily highly expressed in spinal commissural neurons [38], retina [39], and many projection neurons of the forebrain and midbrain during embryonic development [40]. The netrin–DCC interaction can mediate both growth cone attraction and repulsion [37,38,41,42,43,44]. Moreover, Keino-Masu and colleagues discovered that NEO, a transmembrane protein strictly related to DCC, acts as a passive netrin receptor, serving as a stabilizer of the ligand gradient [38].

### 2.5. Slits

Slit is a secreted protein containing leucine-rich and Epidermal Growth Factor (EGF)-like repeats. First discovered in *Drosophila melanogaster* (*D. melanogaster*) by the end of the 1980s, slit is expressed in midline cells and required for normal development of midline structures [45]. Slit proteins are a class of single peptides of approximately 1500 amino acids. Invertebrates have only one slit, while vertebrates harbor three different variants, specifically SLIT1, SLIT2, and SLIT3 [46]. Slit proteins are cleaved by proteolytic enzymes between the fifth and sixth EGF-like domains to generate the long N-terminal Slit segment (SlitN) and the short C-terminal Slit segment (SlitC). These two domains have very different mediators; while SlitN can combine with the main slit interactors, Roundabout (ROBO) and DSCAM, to mediate axon guidance and branching extension, SlitC cannot bind ROBOs [47], but instead regulates axon guidance through its binding to PLEXIN, the main semaphorin receptor [48]. The first ROBO gene, ROBO1, was identified in *D. melanogaster* during an extensive screening focused on genes controlling the CNS midline crossing. [49]. The mammalian ROBO family is composed of four major components (ROBO1–4). While ROBO1 and ROBO2 mediate canonical slit signaling, ROBO3 and ROBO4 exhibit divergent features. ROBO3 cannot bind slits, but instead interacts with the Netrin-1–DCC complex [50]. Moreover, it antagonizes the SLIT2–ROBO1/2-induced repulsion by binding the diffusible factor NELL2. Recently, Pak and colleagues demonstrated the structural interplay between ROBO3 and NELL2, testing in vitro NELL preference towards ROBO3.1 binding [51]. On the other hand, ROBO4 cannot bind to slits directly, but interacts with the complex of SLIT2 and ROBO1 [52]. In addition, it can bind UNC5B, acting as a ligand to inhibit vascular endothelial growth factor (VEGF)-induced angiogenesis and vascular permeability [53]. It has been also proposed that ROBO4 transduces the downstream signaling through the interaction of a co-receptor and other molecules, such as ROBO1, with heparan sulfate proteoglycans (HSPGs) [54].

## 3. Axonal Guidance Cue Integration and Crosstalk

The high degree of complexity in studying axonal wiring is not due to the number of guidance cues, which are rather limited, but is likely derived from the combinatorial effect these cues induce at the growth cone. In fact, the axon guidance cue crosstalk, both in time and space, is fundamental for the proper shaping of axon-related molecular pathways. There are multiple mechanisms implicated in the mediation and regulation of axon guidance-induced responses, from alternative splicing, protein synthesis, and degradation to receptor trafficking and receptor–receptor interactions [55]. Moreover, during their “journey”, axons are often guided by the epistatic influence of intermediate targets, which can switch from a repulsive to an attractive activity. This occurs, for instance, during the triggering of the SLIT/ROBO pathway, which inactivates the netrin-dependent attraction and drives the differential axonal preference in embryonic *Xenopus laevis* commissural spinal neurons [56]. Alternatively, another recent clear-cut example is the extracellular environment-mediated tuning of PLEXIN1a and ROBO1 receptors trafficking on the cell surface during the midline crossing of spinal cord commissural axons in chick embryos [57]. Synergistic crosstalk has also been reported for ephrin and netrin pathways in in vitro explants of chick spinal lateral motor column (LMC) neurons. It has been shown that Ephrin-A5, acting through its receptor EPHA4, induces sensitization to the Netrin-1 signal by increasing NEO abundance in motor neurons, probably acting on the receptor trafficking [58]. Therefore, through a selective combinatorial integration between different classes of axonal cues, a deep fine-tuning of neuronal modular patterning is achieved, allowing for the formation of the complex dynamic network of responses to environmental stimuli that shape the early brain embryonic development. The modular structure of major axonal guidance cue-related receptors and their respective ligands is depicted in Figure 1.

## 4. Lysosomal Function in Axonal Development

To correctly integrate signals coming from extracellular guidance cues, growing axons need to dynamically regulate the presence of receptor proteins available on the growth cone surface. Without considering the transcriptional aspect of this regulation, receptor presence and availability on the cell surface can be post-transcriptionally regulated by the endosomal–lysosomal pathway [59]. Endosomes participate in the dynamics of axonal growth, regulating the trafficking of endocytosed receptors [60]. Once inside the endosomes, receptors can be directed back to the cell membrane if they enter the recycling pathway, or they can be destined to degradation following the late endosomal–lysosomal pathway [61]. Indeed, endosomes can take part in the axonal guidance-related signaling cascades, being the host compartment for sorting signals [59]. For instance, the endo-lysosomal compartment is involved in guidance cue regulation of the EphA2 signaling cascade. In fact, it has been reported that an activated EphA2 receptor can be internalized by trans-endocytosis into endosomes and be recycled back to the plasma membrane or degraded into lysosomes. Moreover, in early endosomes, EphA2 can retain its active state and signal by recruiting and activating the Rac1-specific guanine nucleotide exchange factor (GEF) Tiam1, which seems to be implicated in neurite outgrowth [62,63]. Additionally, in commissural axons, ROBO levels are regulated by lysosomal degradation, thanks to the action of Commissureless (Comm), a late endosomal protein that targets ROBO to late endosomes/lysosomes, allowing the growing axon to cross the midline and reduce its sensibility towards slit-mediated repulsion [64]. The paramount importance of lysosomal function in axonal growth is also highlighted by the fact that inhibiting lysosome transport to the distal axon causes severe changes in size and dynamics of the growth cone [65]. As previously suggested, the impairment of lysosomal trafficking along the axon can affect growth cone homeostasis due to the lack of lysosomal degradative activity or a missing lysosomal-mediated delivery of signaling and adhesion molecules [65]. Moreover, it has been recently shown that RNA granules can also hitchhike on lysosomes to travel long distances in neurons, suggesting that the impairment of lysosomal movement could also imbalance local protein synthesis at the distal axon tip [66]. As a matter of fact, Corradi and colleagues reported that pre-miRNA can travel to the axon terminal, tagging late endosomes/lysosomes, and that SEMA3A signaling induces their maturation with consequent impact on growth cone dynamics [67]. Continuous anterograde transport of degradative active lysosomes and disrupted axon homeostasis due to lysosomal stalling have also been recently described by Farfel-Becker and colleagues, demonstrating that interference with lysosomal transport induces autophagic stress and accumulation of autophagosomes in the axons [7]. Local mitophagy has also been reported to occur in axons after induction of mitochondrial damage [68], further pointing out the relevance of lysosomal regulation and function in axons.

However, local degradation of cargos in axons is not the only mechanism by which lysosomes regulate waste removal and maintain homeostasis. As a matter of fact, retrograde transport of autophagosomes, together with their maturation and fusion with lysosomes, may suggest that the contribution of both local degradation and retrograde transport are mechanisms necessary to obtain efficient axonal clearance [69].

## 5. Brain Disorders with Axonal Guidance Defects

The correct assembly of neural circuits is crucial for cognitive development and interference of axonal growth, and pathfinding has been associated with the onset of neurodevelopmental disorders, such as autism, schizophrenia, and other, more rare conditions. Nonetheless, it has been progressively recognized that also in neurodegenerative disorders (for example, Parkinson’s, Alzheimer’s, and Huntington’s disease), neural circuit impairments precede and carry over the progressive cognitive decline in affected patients [70,71,72,73,74,75]. In some inherited conditions (Table 1), a common pathological feature related to axon trajectories defects is the partial or complete agenesis of the corpus callosum (ACC), a peculiar placental mammalian-specific structure consisting of a large fiber tract that connects the two brain hemispheres [76]. Defects in corpus callosum formation and interhemispheric communication have been demonstrated in autism, schizophrenia, attention deficit hyperactivity disorders, and developmental language disorders [76]. In most cases, failure of contralateral callosal targeting, that is, the impairment of midline crossing and the contralateral positioning of the cortical callosal neurons projections, predispose subtle to gross behavioral abnormalities that severely affect diseased conditions, such as psychiatric disorders. Among identified molecular causes leading to aberrant axonal misrouting and corpus callosum agenesis or dysgenesis, mutations in the chemoattractant ligand netrin have been demonstrated to be detrimental and the leading cause of the congenital mirror movement (CMM) syndrome [77,78]. In these patients, the characteristic feature is synkinesis, that is, an involuntary movement occurring in one side of the body that mirrors intentional movements on the opposite side. This defect can also be diagnosed in patients harboring mutations in the DCC coding gene; in this latter case, partial or total ACC has been described [79,80]. While CMM abnormalities are not generally characterized by intellectual disabilities, the partial or complete ACC may be associated with mild to severe forms of developmental disabilities and cognitive impairment. Perturbed Netrin-1 signaling due to loss-of-function DCC mutations has been also described in the so-called “developmental split-brain syndrome” (DSBS), a severe neurological disease characterized by horizontal gaze palsy, scoliosis, ACC, and midline brain stem cleft [81]. In affected patients, biallelic homozygous mutations have been detected and associated with a complete absence of anterior and hippocampal commissures. Severe neurological abnormalities and intellectual disability have been also ascribed to mutations of the ROBO3 gene in the horizontal gaze palsy and progressive scoliosis (HGPPS) syndrome. In these patients, the cognitive impairment is associated with congenital absence of conjugate horizontal eye movements, preservation of vertical gaze and convergence, and progressive scoliosis developing in childhood and adolescence [82,83,84,85]. X-linked neurodevelopmental forms of intellectual disability have been also described in association with mutations of the L1CAM gene, coding for a neuronal cell adhesion molecule L1, which is involved in axon outgrowth and pathfinding, through interactions with various extracellular ligands and intracellular second messengers [86,87]. Although initially recognized as distinct clinical entities, several congenital forms characterized by L1CAM mutations are now classified as CRASH syndrome (Corpus callosum agenesis, Retardation, Adducted thumbs, Shuffling gait, and Hydrocephalus), a quite heterogeneous group of diseased conditions [88,89]. Aberrant corticospinal tract (CST) development, associated with mirror movement and hypogonadism, is due to defects in the ANOS1 gene in Kallmann syndrome. ANOS1 encodes anosmin, an extracellular glycoprotein important for the axonal guidance and migration of olfactory and Gonadotropin-Releasing Hormone (GnRH) neurons during brain development [90]. The activity of this protein has been largely investigated and has been recently shown to rely on the activation of the fibroblast growth factor (FGF) signaling pathway through a heparan sulfate-dependent mechanism [91]. A characteristic phenotypic feature related to impaired axonal guidance is also the aberrant decussation of nerve fibers. In Joubert syndrome and related disorders (JSRD), a reduced decussation of the superior cerebellar peduncles has been tied to the onset of social disabilities and synkinetic mirror movements [92,93]. The syndrome is associated with defects in several genes (at least 30), most of which play a role in the function of the primary cilium [93]. Among them, the gene ADP-ribosylation factor-like protein 13B (ARL13B) codes a small GTPase, which regulates Sonic Hedgehog (Shh) signaling, and its inactivation results in defective commissural axon guidance in vivo [94]. Another featured example of disorders of misguided axonal branching is the Duane retraction syndrome (DRS), a congenital form of strabismus caused by mutations of the α2-Chimerin [95]. This gene codes for a Rac1 GTPase-activating protein, a cytoskeletal-related protein involved in Ephrin-A-mediated spine morphogenesis [96] and required for oculomotor axon guidance targeting [97,98]. Dysgenesis of the corpus callosum and anterior commissure have been identified in patients harboring mutations in the TUBB3 gene, which cause two distinct clinical entities, named Cortical Dysplasia, Complex, with other Brain Malformations 1 (CDCBM1) and Congenital Fibrosis of the Extraocular Muscles 3 (CFEOM3) [98,99]. In both cases, the loss of function occurring in the third (III) member of the beta-tubulin protein family (TUBB3) leads to microtubule instability and axonal guidance defects in commissural axons and cranial nerves that result in intellectual and behavioral impairments and aberrant eye movement [100]. Additional *corpus callosum* defects, although with minimal or undetectable intellectual disability, have been described in the Craniofrontonasal syndrome (CFNS), in which loss-of-function Ephrin-B mutations primarily affect the boundaries of the coronal cranial suture, leading to pathological craniosinostosis [101]. While no direct evidence of abnormal decussation or impaired commissures formation has been detected in patients affected by lysosomal storage disorders, recent investigations have suggested the potential implication of axonal guidance defects in the onset of neurological abnormalities in Mucopolysaccharidosis (MPS) type II (Hunter syndrome), type IIIb (Sanfilippo syndrome), and type VII (Sly syndrome) [102,103,104]. In MPSII and MPSIIIb, the aberrant heparan sulfate catabolism associated with the onset of progressive severe neurological abnormalities have been tied to neuronal dysfunction and misexpression of axonal guidance cues [102,103]. Future studies will enable us to verify whether the cognitive decline observed in these and other LSD diseases is tightly related to axonal guidance-related abnormalities.

## 6. Concluding Remarks

In light of the recent discoveries, the contribution of lysosomes to the process of axonal guidance and remodeling has gained substantial interest. The utmost importance of correct lysosomal hydrolases activity and, more in general, of lysosomal trafficking and function, pinpoints and justifies increasing research efforts towards the study of these organelles in the context of neurological disorders. Bearing in mind that lysosomal storage disorders often exhibit severe neurological abnormalities, starting from early childhood, it appears to be groundbreaking in the investigation of the functional relationship between axonal guidance and lysosomal protein activity. A more detailed understanding of this hypothetical epistatic interaction would encourage the development of more targeted therapies against neurological defects in both lysosomal disorders and neurodegenerative conditions.

## Figures and Tables

**Table 1:** Brain disorders in which axon guidance alterations have been described or inferred.

Disorder	Human Gene	Axon Guidance-Related Defect	Symptoms	Reference
Congenital mirror movements (CMM) and partial or complete agenesis of the *corpus callosum*	NETRIN1 (NTN1)	Corticospinal tract (CST) abnormality	Involuntary movements of one hand that mirror intentional movements of the opposite hand	[77,78]
Congenital mirror movements (CMM) and/or isolated agenesis of the *corpus callosum*	Deleted in colorectal cancer (DCC)	Decreased crossing of descending corticospinal tract projections	Variable range of intellectual disabilities, cognitive impairment, language delay, and visual and spatial deficits	[79,80]
Developmental split-brain syndrome (gaze palsy, familial horizontal, with progressive scoliosis 2, with impaired intellectual development)	Deleted in colorectal cancer (DCC)	Agenesis of the *corpus callosum* and absence of the anterior and hippocampal commissures	Neurological abnormalities, horizontal gaze palsy, intellectual disability, and progressive scoliosis	[81]
Horizontal gaze palsy with progressive scoliosis (HGPPS)	Roundabout guidance receptor 3 (ROBO3)	Abnormal flattening of the basis pontis and hypoplasia in the pontine tegmentum; anomalous innervations of the lateral rectus muscle of the eye by the abducens supranuclear nerve	Horizontal gaze palsy, intellectual disability and progressive scoliosis	[82,83,84,85]
CRASH syndrome (*Corpus callosum* agenesis, Retardation, Adducted thumbs, Shuffling gait, and Hydrocephalus)	L1CAM	Agenesis of the *corpus* *callosum* and corticospinal tract	Microcephaly, mental retardation, spastic paraparesis	[86,87]
Kallman syndrome (X-linked)	ANOS1 (KAL1)	Defective olfactory axon guidance and migration	Congenital anosmia, hypogonadotropic hypogonadism, mirror movements, and aberrant corticospinal tract	[89,90]
Joubert syndrome and related disorders (JSRD)	Multiple genes (AHI1, NPHP1, CEP290, TMEM67, RPGRIP1, ARL13B, CC2D2A)	Hypotonia, ataxia, mental retardation, altered respiratory patterns, social disabilities, and synkinetic mirror movements	Cerebellar vermian hypoplasia, reduction in pontine neurons, and reduced decussation of the superior cerebellar peduncles.	[92,93]
Duane retraction syndrome (DRS)	α2-CHIMERIN	Absence of *abducens* motor neurons and nerves; aberrant innervation of the lateral rectus muscle by the oculomotor nerve	Restricted horizontal gaze and ocular synkinesis	[95,96]
Cortical dysplasia, complex, with other brain malformations 1 (CDCBM1)	Beta tubulin protein family member TUBB3	Thin *corpus callosum*, hypoplastic brainstem, and dysplastic cerebellar vermis	Severe mental retardation, strabismus, axial hypotonia, and spasticity	[99]
Congenital fibrosis of the extraocular muscles 3 (CFEOM3)	Beta tubulin protein family member TUBB3	Dysgenesis of the *corpus callosum* and anterior commissure (AC), and internal capsule; generalized loss of white matter; basal ganglia dysmorphisms	Aberrant eye movements, facial weakness, axonal peripheral neuropathy, contractures of the wrist and fingers, delayed development, and learning disabilities	[100]
Craniofrontonasal syndrome (CFNS)	Ephrin B1(EFNB1)	Dysgenesis or agenesis of the *corpus callosum*	Variable difficulties in speech and language, limited or no intellectual disabilities, facial asymmetry, skeletal and dermatological abnormalities	[101]
Mucopolysaccharidosis type II (Hunter syndrome)	Iduronate sulfatase (IDS)	Indirect experimental observation	Mental retardation, language delay, cognitive impairment	[103]
Mucopolysaccharidosis type IIIb (Sanfilippo Syndrome)	α-N-acetylglucosaminidase (NAGLU)	Indirect experimental observation	Mental retardation, cognitive decline, dysphagia, sleep problems, seizures	[102]
Mucopolysaccharidosis type VII (Sly syndrome)	β-glucuronidase (GUSB)	Indirect experimental observation	developmental Delay, speech delay, intellectual disability of variable degree	[104]
